# Chemerin15-Ameliorated Cardiac Ischemia-Reperfusion Injury Is Associated with the Induction of Alternatively Activated Macrophages

**DOI:** 10.1155/2015/563951

**Published:** 2015-06-16

**Authors:** Chao Chang, Qingwei Ji, Bangwei Wu, Kunwu Yu, Qiutang Zeng, Shuanli Xin, Jixiang Liu, Yujie Zhou

**Affiliations:** ^1^Department of Cardiology, Beijing Anzhen Hospital, Capital Medical University, Beijing Institute of Heart, Lung and Blood Vessel Disease, The Key Laboratory of Remodeling-Related Cardiovascular Disease, Ministry of Education, Beijing 100029, China; ^2^Institute of Cardiovascular Diseases, Union Hospital, Tongji Medical College, Huazhong University of Science and Technology, Wuhan 430022, China; ^3^Department of Cardiology, Handan First Hospital, Handan 056002, China

## Abstract

Chemerin15 (C15), an endogenous anti-inflammatory component, inhibits the activity of neutrophils and macrophages through G protein-coupled receptor ChemR23; however, its role as well as functional mechanism in mouse myocardial ischemia/reperfusion (I/R) injury remains unknown. *Methods.* Sham or I/R operations were performed on C57BL/6J mice. The I/R mice received an injection of C15 immediately before reperfusion. Serum troponin T levels, infarct size, cardiomyocyte apoptosis, reactive oxygen species (ROS) production, and infiltration of neutrophils were assessed 24 h after reperfusion, while the macrophage phenotypes, macrophage infiltration, and inflammatory cytokine levels were assessed 48 h after reperfusion. *Results.* Compared with the control group, the C15-treated mice showed an obvious amelioration of I/R injury and displayed less ROS, accompanied by reduced neutrophil recruitment. C15 decreased the tumor necrosis factor- (TNF-) *α* and interleukin- (IL-) 6 levels and increased the IL-10 levels in the serum of the I/R mice, which suggested a suppressed inflammatory response that could be related to elevated alternatively activated M2 macrophages with characteristic skewed expression of M2 markers and inhibition of classically activated M1 marker expression. *Conclusion.* C15 may induce alternatively activated M2 macrophage polarization and suppress the inflammatory response to protect against myocardial I/R injury in mice.

## 1. Introduction

Myocardial ischaemia/reperfusion (I/R) injury, an inflammatory condition, is characterized by the rapid influx of leukocytes, followed by cytokine secretion, increased oxidative stress, and protease release in the endangered myocardial region [1, 2]. Prolonged ischemia and excessive inflammatory responses after I/R are deleterious for cell survival and extracellular matrix integrity, resulting in irreversible cell apoptosis or necrosis, which further amplifies the inflammatory response and provokes a positive feedback loop to aggravate I/R injury [1, 3, 4]. Thus, interventions targeting leukocytes or inflammatory mediators could significantly ameliorate myocardial I/R injury [2, 5].

Improper macrophage activation is pathogenically related to various metabolic and immune-mediated inflammatory disorders, including myocardial I/R injury [6, 7]. Macrophages express distinct patterns of surface receptors and metabolic enzymes in response to different stimuli, which ultimately results in diversified macrophage functions and phenotypes [8]. Broadly, two distinct polarization states of macrophages have been characterized, namely, M1 and M2 [9]. Classically activated M1 macrophages display a proinflammatory profile, whereas alternatively activated M2 macrophages exhibit anti-inflammatory and tissue repair properties. M2 macrophages play a significant role in the immune system by preventing an excessive inflammatory response, which has been proven to be protective in various autoimmune diseases [9, 10]. These M2 macrophages show modulation of the expression of cell surface receptors, leading to enhanced endocytic and phagocytic activities [11, 12]. Methods limiting M1 while promoting M2 polarization of macrophages could represent a unique therapeutic strategy to suppress inflammatory responses and ameliorate myocardial I/R injury [7, 13, 14].

Chemerin, a new member of the adipokine family, is highly expressed in placenta, liver, and white adipose tissues. To a lesser extent, chemerin is also expressed in many other tissues, such as the lungs, brown adipose tissue, heart, ovaries, kidneys, skeletal muscle, and pancreas. Acting through the receptor ChemR23, chemerin functions as a chemoattractant for neutrophils, macrophages, immature dendritic cells (DCs), and natural killer** (**NK) cells [15–18]. Circulating chemerin levels are elevated in numerous diseases associated with chronic inflammation, such as Crohn's disease, liver disease, psoriasis, arthritis, lupus, and atherosclerosis, which suggests that chemerin may play a proinflammatory role in autoimmune diseases [18–22]. Inactive prochemerin is converted to the bioactive chemerin isoform through proteolytic removal of the last 6 amino acids at its C-terminal by neutrophil-derived proteases (elastase and cathepsin G), mast cell products (tryptase), and proteases of the coagulation cascade [23, 24]. One previous study demonstrated that the calpains and cathepsin S derived from activated macrophages can cleave chemerin to generate the potent anti-inflammatory peptide chemerin15 (C15, A140–A154). C15 exhibits obvious anti-inflammatory properties, although its chemoattractant activity is reduced [25]. In a murine cutaneous excisional wound model, C15 promoted tissue repair and reduced scarring by dampening the immediate intravascular inflammatory response, including inhibition of leukocyte recruitment and macrophage activation [26]. In addition, a recent study showed that C15 reduced neutrophil recruitment and heart damage in a murine myocardial infarction model [27]; however, the mechanism for this effect has not been investigated.

In the present study, we hypothesized that C15 may induce M2 polarization, which would lead to further inhibition of the inflammatory response as well as decreased cardiomyocyte apoptosis and enhanced cardiac function after myocardial I/R.

## 2. Materials and Methods

### 2.1. Animals and Experimental Design

Male C57BL/6 mice (8–10 weeks of age) were purchased from the Beijing University (Beijing, China). The mice were fed a standard diet and given water* ad libitum* at the Animal Care Facility of Capital Medical University, according to the institutional guidelines. The mice underwent sham or I/R surgery. The present study included three experimental groups: sham, control I/R, and I/R + C15 (each group contained six to eight mice). The treatment group received C15 (0.3 ng/kg dissolved in 200 *μ*L normal saline) (GL Biochem, China) through the tail vein before reperfusion [27]. In addition, C15 was repeatedly administered every 12 h over the 48 h reperfusion period. The serum troponin T levels and the infarct size were measured 24 h after reperfusion, and cardiac functions were assessed 48 h after reperfusion. Cell apoptosis, reactive oxygen species (ROS) production, and neutrophil infiltration were assessed 24 h after reperfusion, while macrophage phenotypes, macrophages infiltration, and inflammatory cytokine levels were measured 48 h after reperfusion [28]. All animal experiments were approved by the Animal Care and Utilization Committee of Capital Medical University, China.

### 2.2. Myocardial I/R Injury* In Vivo* and Infarct Area Assessment

The murine I/R model was performed as previously described [29]. The mice were anaesthetized by an intraperitoneal injection of pentobarbital sodium (50 mg/kg). The mice were orally intubated and then connected to a rodent ventilator. A left thoracotomy was performed through a horizontal incision at the third intercostal space. I/R was achieved by ligating the anterior descending branch of the left coronary artery with 8-0 silk suture, and silicon tubing (PE-10) was placed on top of the left anterior descending (LAD) coronary artery, 2 mm below the border between the left atrium and left ventricle (LV). Ischemia was confirmed by an electrocardiogram (ST elevation). The sham-operated animals were subjected to the same surgical procedures, except that the suture was passed under the LAD coronary artery but not tied. After 45 min of occlusion, the silicon tube was removed to achieve reperfusion, and the rib space and overlying muscles were closed. Echocardiographic analyses were performed before euthanasia. Twenty-four hours after reperfusion, the animals were reanesthetized, the heart function was blocked at the diastolic phase by administering potassium chloride (20 mM) injection, the chest was opened, and the ascending aorta was cannulated and perfused with saline to wash out the blood. The LAD coronary artery was occluded with the same suture, which had been left at the site of ligation. To demarcate the ischemic area at risk (AAR), Alcian blue dye (1%) was perfused into the aorta and coronary arteries. The hearts were excised, and the LVs were sliced into 1 mm thick cross sections. The heart sections were then incubated with a 1% triphenyltetrazolium chloride solution at 37°C for 15 min. The infarct area (pale), AAR (not blue), and total LV area from both sides of each section were measured using Image-Pro Plus software (Media Cybernetics, Inc., United Stated of America (USA)); the obtained values were averaged. The percentages of the area of infarction and AAR of each section were multiplied by the weight of the tissue section and then summed for all sections.

### 2.3. Serum Troponin T

The serum troponin T levels were measured as an index of cardiac cellular damage using a Quantitative Rapid Assay Kit (Roche Diagnostics GmbH, Germany).

### 2.4. Evaluation of Apoptosis in Tissue Sections

Deoxyribonucleic acid (DNA) fragmentation was detected* in situ* using terminal deoxynucleotidyl transferase-mediated dUTP nick end labeling (TUNEL), as previously described [30]. The excised hearts were fixed in 4% paraformaldehyde, embedded in paraffin, cut into 5 *μ*m thick sections, and treated as instructed in the In Situ Cell Death Detection Kit (Roche Diagnostics GmbH, Germany). The cardiac caspase-3 activity was measured using a Caspase-3 Colorimetric Assay Kit, according to the manufacturer's instructions (Chemicon Pvt. Ltd., USA). The absorbance of 4-nitroaniline cleaved by caspase-3 was measured at 405 nm using a microplate reader (ELx800, BioTek Instruments, USA).

### 2.5. Lipid Peroxidation Detection

The ischemic zones were assessed for lipid peroxidation as an indicator of ROS activity. The supernatants of homogenized cardiac tissues were analyzed for lipid peroxidation products (malondialdehyde (MDA)/4-hydroxyalkenals) using a Lipid Peroxidation Assay Kit (Calbiochem, Germany).

### 2.6. Myeloperoxidase (MPO) Detection

Neutrophils infiltration was assessed with a Myeloperoxidase Activity Colorimetric Assay Kit (Biovision, USA) by measuring the MPO activity in the ischemic myocardium, according to the manufacturer's instructions.

### 2.7. Immunohistochemical Analysis

The cardiac tissues were fixed with 4% formalin solution, embedded in paraffin, and sectioned into 6 mm thick slices. To investigate macrophage infiltration after I/R injury, the sections were stained with the anti-mouse macrophage marker F4/80 (BD Biosciences, USA), following standard protocols.

### 2.8. Enzyme-Linked Immunosorbent Assay (ELISA) for Proinflammatory Factors

The serum levels of tumor necrosis factor- (TNF-) *α*, interleukin- (IL-) 6, and IL-10 were measured using a commercially available ELISA Kit (R&D Systems, USA). All procedures were performed twice, and the results were reported in pg/mL.

### 2.9. Flow Cytometric Analysis

Forty-eight hours after reperfusion, the ischemic zones of cardiac tissues from I/R mice were obtained to analyze macrophage infiltration and phenotype using the following fluorochrome-labeled antibodies: allophycocyanin- (APC-) conjugated anti-mouse F4/80, phycoerythrin- (PE-) conjugated anti-mouse inducible nitric oxide synthase (iNOS), and PE-Cyanine7-conjugated anti-mouse CD206 (eBiosciences, USA).

### 2.10. Real-Time Polymerase Chain Reaction (PCR)

Total ribonucleic acid (RNA) was extracted from the cardiac tissues using TRIzol reagent (Invitrogen, USA) and reverse-transcribed into complementary DNA using the PrimeScript RT Reagent Kit (Takara Biotechnology, China), according to the manufacturer's instructions. The messenger RNA levels of the target genes were quantified using SYBR Green Master Mix (Takara Biotechnology), with the ABI PRISM 7900HT Sequence Detector System (Applied Biosystems, USA). Each reaction was performed twice, and the changes in the relative gene expression, which were normalized to glyceraldehyde 3-phosphate dehydrogenase levels, were determined using the relative threshold cycle method. The primer sequences are shown in Table 1.

### 2.11. Statistical Analysis

The data were presented as the means ± SEM. The differences between the two groups were evaluated using an unpaired Student's *t*-test. One-way analysis of variance (ANOVA) was used for multiple comparisons, followed by Tukey's post hoc procedure for multiple range tests. All statistical analyses were performed using SPSS software, version 17.0 (SPSS, Inc., USA). A *P* value less than 0.05 was considered statistically significant.

## 3. Results

### 3.1. C15 Alleviated Myocardial I/R Injury in Mice

**Figure 1:**
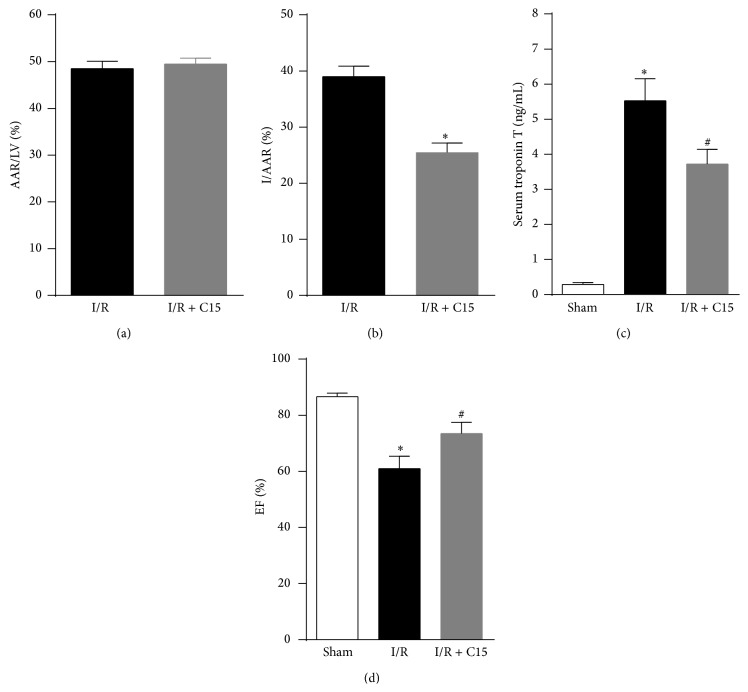
Chemerin15 (C15) decreased the myocardial infarct size and improved the hemodynamic performance following ischemia/reperfusion (I/R). ((a) and (b)) Quantification of the area at risk/left ventricular area (AAR/LV) and infarct area/AAR (I/AAR) 24 h after reperfusion (*n* = 6). (c) Serum cardiac troponin T (cTnT) was measured in the sham, I/R, and I/R+C15 groups at 24 h after I/R (*n* = 5). (d) The ejection fraction (%EF) was measured 48 h after reperfusion (*n* = 6). *∗* < 0.05 versus the I/R or sham groups; # < 0.05 versus the I/R group.

The AAR : LV ratio did not differ significantly between the C15-treated and untreated groups (Figure 1(a)), suggesting that the ligation was reproducibly performed on the same LAD level. In the I/R mice, the administration of C15 decreased the infarct size within the AAR by 35.2% compared to the untreated group (Figure 1(b)). Similarly, the serum level of cardiac troponin T, a direct index of myocyte damage, was also significantly lower in the C15-treated group (Figure 1(c)). As shown in Figure 1(d) (by echocardiography), 48 h after I/R, the ejection fraction (EF) was significantly reduced in the I/R mice compared to the sham group. However, in the C15-treated group, high EF was observed at 48 h after reperfusion, indicating an obvious alleviation of I/R-induced cardiac dysfunction. These results showed that C15 could protect the heart against myocardial I/R injury in mice.

### 3.2. C15 Reduced Cardiomyocyte Apoptosis and Neutrophil Infiltration

**Figure 2:**
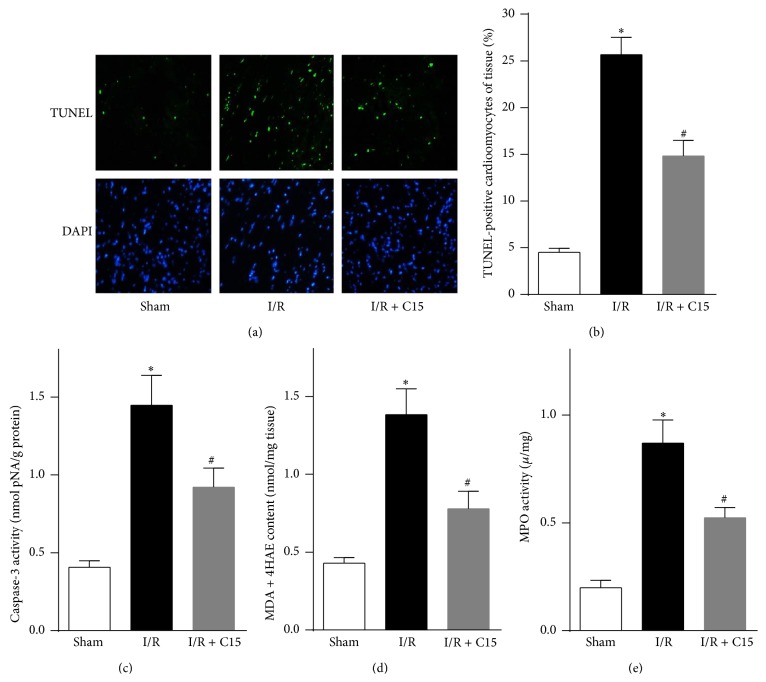
C15 reduced cardiomyocyte apoptosis and neutrophil infiltration. (a) Representative photographs of terminal deoxynucleotidyl transferase-mediated dUTP nick end labeling- (TUNEL-) stained ischemic area 24 h after reperfusion. For these images, apoptotic nuclei are indicated by TUNEL staining (green), and total nuclei are indicated by 4,6-diamidino-2-phenylindole (DAPI) staining (blue); bar, 10 *μ*m. (b) Quantitative analysis of TUNEL-positive nuclei over the total number of nuclei* in vivo* (*n* = 6). (c) Caspase-3 activity in the ischemic myocardium was assessed after 24 h of reperfusion (*n* = 5). (d) Malonaldehyde+4-hydroxy-alkenals (MDA+4HAE) contents in the ischemic myocardium were evaluated using a lipid peroxidation assay (*n* = 5). (e) Cardiac myeloperoxidase (MPO) activity was detected in ischemic myocardia (*n* = 6). *∗* < 0.05 versus the sham group; # < 0.05 versus the I/R group.

Cell apoptosis has been proposed to play an important role in myocardial I/R injury [31]. Compared with control mice, C15-treated I/R mice showed fewer apoptotic cells in the ischemic myocardium, as demonstrated by TUNEL staining (Figures 2(a) and 2(b)). Moreover, the C15 treatment significantly suppressed the activation of caspase-3 in the ischemic myocardium of the I/R mice (Figure 2(c)). Furthermore, C15 treatment resulted in decreased neutrophil recruitment, as demonstrated by less MPO activity in the ischemic myocardium (Figure 2(e)). Moreover, the level of myocardial lipid peroxidation was significantly decreased in the C15-treated I/R mice compared with the control myocardium of I/R mice (Figure 2(d)). These results suggest that C15 may prevent cardiomyocyte apoptosis by inhibiting neutrophil activity.

### 3.3. C15 Generated Alternatively Activated Macrophages in the Ischemic Myocardium

**Figure 3:**
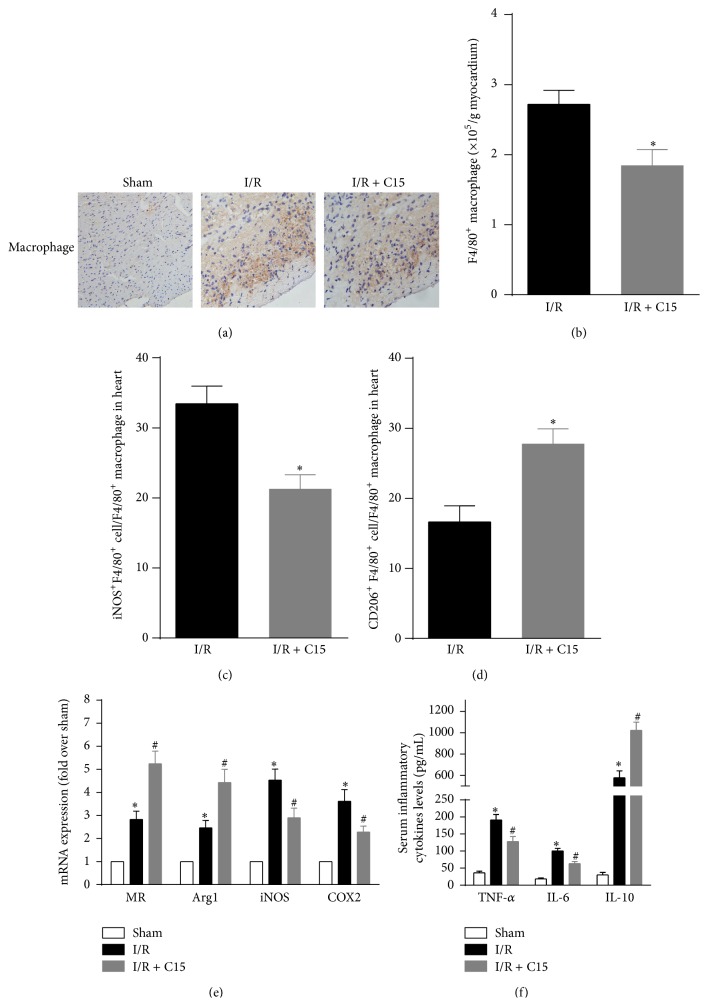
C15 generated alternatively activated macrophages in the ischemic myocardium. (a) Representative photographs of macrophage infiltration in the ischemic myocardium after 48 h of reperfusion. (b) The number of F4/80^+^ macrophages infiltrated into the ischemic myocardium was analyzed by flow cytometry (*n* = 6). (c) The number of classically activated M1 (iNOS^+^F4/80^+^) macrophages infiltrated into the ischemic myocardium was analyzed with flow cytometry (*n* = 6). (d) The number of alternatively activated M2 (CD206^+^F4/80^+^) macrophages that were infiltrated into the ischemic myocardium was analyzed with flow cytometry (*n* = 6). (e) M1 and M2 markers were analyzed with real-time PCR (*n* = 6). (f) The serum levels of proinflammatory cytokines (tumor necrosis factor-*α* (TNF-*α*) and interleukin-6 (IL-6)) and anti-inflammatory cytokines (IL-10) were measured after 48 h of reperfusion (*n* = 6). *∗* < 0.05 versus the I/R or sham groups; # < 0.05 versus the I/R group.

Our results showed that C15 could significantly reduce macrophage infiltration in the ischemic myocardia of I/R mice (Figures 3(a) and 3(b)). To determine the effect of C15 treatment on macrophage polarization, the proportion of CD206^+^ cells among macrophages was measured. The C15-treated I/R mice displayed higher percentages of CD206^+^F4/80^+^ M2 cells compared with the control group (Figure 3(d)), while the percentage of iNOS^+^F4/80^+^ M1 cells was decreased (Figure 3(c)). Furthermore, the C15 treatment increased the expression of M2 markers, including arginase 1 (Arg1) and mannose receptor (MR), and inhibited the expression of M1 markers, including iNOS and cyclooxygenase-2 (COX-2) (Figure 3(e)), which was consistent with the flow cytometry results.

These results led us to hypothesize that the suppression of macrophage M1 activation by C15 may have led to changes in inflammatory cytokine expression. As shown in Figure 3(f), I/R increased the serum levels of TNF-*α* and IL-6, whereas C15 treatment resulted in significant suppression of the abovementioned proinflammatory cytokines. Furthermore, the level of IL-10 (an anti-inflammatory cytokine) was markedly increased in C15-treated I/R mice compared to control mice. Therefore, C15 may promote macrophage M2 polarization and thus maintain immune tolerance and confer protection against myocardial injury.

## 4. Discussion

The primary aim of the present study was to examine the role of C15 in mouse myocardial I/R injury and the protective mechanism of the actions involved in that process. Our results showed that C15 treatment clearly reduced the infarct size and improved cardiac function, and these results were accompanied by decreased cardiomyocyte apoptosis and neutrophil infiltration. Furthermore, C15 promoted alternative M2 macrophage activation and suppressed the proinflammatory response.

It is well known that a vigorous inflammatory response, mediated by several types of infiltrating leucocytes and related inflammatory cytokines, plays an important role in the pathogenesis of myocardial I/R injuries [1, 2]. Recruited neutrophils and activated macrophages induce the expression of proinflammatory cytokines and increase both oxidative stress and protease release, which directly exacerbate the myocardial and endothelial injury [3, 7]. Chemerin can stimulate pro- or anti-inflammatory responses, depending on the different pathophysiological conditions of various diseases [18]. Chemerin acts as a chemotactic factor for ChemR23-expressing leukocyte populations, particularly macrophages, immature DCs, and NK cells, to promote their recruitment to localized sites of inflammation and tissue damage in conditions such as immunoglobulin E-mediated anaphylaxis, multiple sclerosis, and experimental autoimmune encephalomyelitis [19, 21, 32]. However, in mouse models of zymosan-induced peritonitis, lipopolysaccharide-induced acute lung inflammation, and excisional cutaneous skin wounds, the inhibition of endogenous chemerin activity or a loss of ChemR23 expression led to the exacerbation of inflammation and increased leukocyte infiltration, suggesting a protective role in these physiologic contexts [16, 25, 26]. Because neutrophils are typically the first cells to arrive at sites of inflammation [33], they can secrete serine proteases, predominantly cathepsin G, and elastase, which are responsible for the proteolytic cleavage of prochemerin to activated chem21–156 or chem21–157, respectively [24]. One previous study demonstrated that calpains and cathepsin S derived from activated macrophages could cleave chemerin to generate the potent anti-inflammatory peptide C15 (A140–A154), which could then inhibit macrophage activation and inhibit their chemotactic activity [25]. Furthermore, C15 was shown to suppress the activation of integrins, which mediate neutrophil adhesion and transendothelial migration* in vitro* [27]. Direct administration of C15 restricted inflammatory cell recruitment to the wound site and improved wound repair in a mouse model of excisional cutaneous wounds [26]. In a zymosan-induced peritonitis model, intraperitoneal administration of C15 demonstrated a significant protective role, as shown by decreased neutrophil and monocyte recruitment as well as reduced proinflammatory cytokine expression [25]. Consistent with the findings of Cash et al. [27], the present study results also showed that C15 suppressed the infiltration of neutrophils and macrophages as well as inflammatory cytokine expression (TNF-*α*, IL-6), which may be related to decreased ROS production, cardiomyocyte apoptosis, and improved cardiac function in mouse myocardial I/R injuries [3, 31]. However, there was some disparity between reports in the extent to which the inflammatory response was inhibited and the protective effect mediated by C15. Nevertheless, these results suggest that the generation of C15 after proteolytic cleavage may function as a negative feedback mechanism to modulate an excessive inflammatory response by suppressing leucocyte infiltration and activation [25–27, 34]. In addition, C-terminal-truncated chemerin variants displayed greater chemotactic or anti-inflammatory effects, which was the result with the cleavage at distinct sites by different classes of proteases [18, 23]. Thus, it could be speculated that the inconsistent role of chemerin between studies may derive from the existence of different classes of proteases in different disease conditions, which produce special levels of chemerin variants, leading to pro- or anti-inflammatory effects [18, 23–25].

The inflammatory response mediated by macrophages plays an important role in myocardial I/R injury [6, 7]. In the present study, it was demonstrated that C15 could modulate macrophages toward an alternative M2 polarization state. In C15-treated myocardial I/R mice, the level of F4/80^+^CD206^+^ M2 macrophages in the ischemic myocardium was clearly higher than that in control I/R mice, which was accompanied by increased expression of M2 markers, including MR and Arg1, whereas the expression of M1 markers, including iNOS and COX-2, was reduced. Macrophages exhibit heterogeneous functions, and their properties and activation state are modulated by local environmental stimuli [9, 11]. Inadequate macrophage activation results in metabolic, inflammatory, and immune disorders that are pathogenically related to various autoimmune diseases [9, 10]. Proinflammatory cytokines (interferon-*γ*, TNF-*α*, and IL-6) induce macrophages towards “classically activated” M1 polarization, and these cells produce proinflammatory mediators, ROS, and proteases, resulting in damage to myocardial and endothelial cells in I/R mice [7, 35]. Conversely, T helper 2 cell-related cytokines, such as IL-4 or IL-13, can polarize macrophages towards an alternatively activated M2 phenotype associated with high levels of anti-inflammatory cytokines (IL-10, tumor growth factor-*β*) and the repair or remodeling of damaged tissues [9, 10]. Cash et al. showed that C15 significantly enhanced macrophage phagocytosis of microbial particles and apoptotic cells* in vitro* and* in vivo* [34]. Apoptotic cell phagocytosis plays an important role in the resolution of inflammation and maintaining immune tolerance. Nevertheless, insufficient clearance of apoptotic cells leads to the accumulation of secondary necrotic cells, which can stimulate the inflammatory response because necrotic cell lysis releases cytotoxic and proinflammatory substances [36, 37]. In autoimmune diseases, such as systemic lupus erythematosus, atherosclerosis, and diabetes mellitus, there is a persistent state of inflammation that may be related to the inefficient clearance of apoptotic cells and the resolution of inflammation [38, 39]. Similar to other adipokines, adiponectin primes human monocyte differentiation into anti-inflammatory M2 macrophages through its ability to promote the clearance of apoptotic cells; during this process, adiponectin serves as a soluble bridge molecule between motifs on the surface of apoptotic cells and the phagocytic receptors on the surface of macrophages. Similarly, C15 may act in concert with prophagocytic signals, including Annexin A1 and Annexin-derived peptides, which are released by apoptotic cells to promote macrophage clearance [34, 40, 41]. In a mouse excisional cutaneous wound model, C15-treated mice expressed lower levels of TNF-*α* and iNOS (typically associated with an M1 phenotype) and higher levels of Arg1 (typically associated with an M2 phenotype) [26]. Furthermore, in C15-treated mice, macrophages appeared to have many protrusions mediated by actin cytoskeleton changes, which may be consistent with the report by McWhorter, who showed that modulation of macrophage shapes could affect macrophage polarization status [26, 42]. Collectively, these data indicate that C15 can skew macrophage towards alternative M2 polarization, thereby inhibiting the inflammatory response and leading to a protective role in heart damage after I/R in a mouse model.

The mechanism through which C15 functions remains unclear. Earlier studies showed that C15 suppressed neutrophil migration and macrophage activation through a mechanism that was dependent on ChemR23* in vitro* and* in vivo *[25, 27], which may seem to contradict the proinflammatory role of full-length chemerin. In addition to C15, ChemR23 has also been shown to be activated by lipid RvE1 to exert anti-inflammatory effects in animal models of zymosan-induced peritonitis and sulfonic acid-induced colitis [43–45]. Thus, ChemR23 may be a multifunctional receptor that transduces proinflammatory and anti-inflammatory effects, and further investigation is needed to determine whether individual chemerin isoforms show differential bioactivity through one particular signaling pathway [18].

Together, the present study results show that C15 can reduce myocardial I/R injury in mice, as demonstrated by decreased cardiomyocyte apoptosis and improved heart function, which may be linked to suppressed proinflammatory responses mediated by alternative M2 macrophages. Thus, our results suggest that C15 may represent a new therapeutic class for the treatment of inflammatory diseases.

## Figures and Tables

**Table 1:** Primer sequences for real-time PCR.

Gene	Forward (5′-3′)	Reverse (5′-3′)
MR	ATGCCAAGTGGGAAAATCTG	TGTAGCAGTGGCCTGCATAG
Arg1	CGCCTTTCTCAAAAGGACAG	ACAGACCGTGGGTTCTTCAC
iNOS	CACCTTGGAGTTCACCCAGT	ACCACTCGTACTTGGGATGC
COX-2	GCGACATACTCAAGCAGGAGCA	AGTTGGTAACCGCTCAGGTGTTG

Arg1, arginase 1; iNOS, inducible nitric oxide synthase; COX-2, cyclooxygenase-2.
